# An eco-friendly smartphone based HPTLC method *versus* conventional densitometric one for determination of Naltrexone and Bupropion

**DOI:** 10.1186/s13065-024-01285-1

**Published:** 2024-09-23

**Authors:** Eman M. Moaaz, Ezzat M. Abdel-Moety, Mamdouh R. Rezk, Ahmed S. Fayed

**Affiliations:** Analytical Chemistry Department, Faculty of Pharmacy, Cairo University, Kasr El-Aini Street, Cairo, ET-11562 Egypt

**Keywords:** Color Picker, ImageJ, Naltrexone-Bupropion, Smartphone applications, White Chemistry

## Abstract

The rapid uprising technologies of smartphone applications and software introduced a new era for analytical detection techniques. It has transformed bench-top laboratory methods into simpler ones depending on cost-effective, portable, and widely accessible devices. In this work, two high performance thin layer chromatographic (HPTLC) methods were developed based on smartphone’s camera detection and either ImageJ desktop software or Color-Picker smartphone’s application as alternative techniques to conventional densitometric detection. A mixture of Naltrexone hydrochloride (NAL) and Bupropion hydrochloride (BUP) was chromatographed on HPTLC- plates using ethyl acetate, methanol, acetone, and glacial acetic acid (3:6:1:0.5, by volume) as a developing system. The developed plates were scanned at 203 nm for the densitometric analysis, then visualized by modified Dragendorff’s reagent and shot by a smartphone’s camera. The captured images were uploaded to either ImageJ software or Color-Picker application to detect the separated spots. The results derived from the three detection methods were compared over the concentration range of 0.4–24 & 0.6–18 µg/band for the densitometric method, 0.4–24 & 2–24 µg/band for ImageJ built method and 0.8–20 & 5–20 µg/band for Color Picker built method for NAL and BUP, respectively. The methods were found to be appropriate for assaying both active drug substances in pure forms and combined in marketed pharmaceutical formulations. The excellent sustainability of densitometric and ImageJ-based methods enabled also the assessment of their dosage form content uniformity. The greenness and sustainability of the methods were assessed by three metric tools, namely Green Analytical Procedure Index (GAPI), Analytical GREEnness Metric Approach (AGREE), and White Analytical Chemistry (WAC). The assessments results confirmed the sustainability and superiority of the proposed methods in terms of sample treatment, waste mount, energy consumption, cost, and number of analyzed samples per an hour.

**Supplementary Information:**

The online version contains supplementary material available at 10.1186/s13065-024-01285-1.

## Introduction

Decades ago, mobile phones have taken a wide part in our daily lives. With rising technologies, they have been converted into smart devices almost inseparable from our hands. Holding a portable smart device with multiple abilities and applications could be useful and save time. Coupling smartphone applications with instrumental analysis has been introduced for some analytical methods, operating as detectors by themselves or linked to other devices or softwares [1]. The strategy of the presented work offered a simple, accessible, and economical alternative to conventional techniques, especially for developing countries during the existing global economic crisis. The combination of thin layer chromatography (TLC) and smartphone applications displaced gradually the traditional densitometry. The main idea was to capture images of the developed plates with a smartphone’s camera after illuminating the plates with an optimum visualizer, followed by performing image analysis by chemometric operations [2], available computer softwares [3–5], and/or mobile phone applications [6]. Some image processing softwares were complicated, inflexible, and intolerant to multiple image processing, on the contrary, ImageJ software was considered to be a user-friendly interface, that could implement various image processing operations and run on any operating system [7]. ImageJ was available in the public domain of the National Institute of Health (NIH) [8], it has been downloaded thousands of times since the launch of the first version back in 1997 and was successfully used for numerous diagnostic, biological, and analytical approaches [9]. In addition, smartphone applications could offer another manner for image analysis; one of these recently applied programs was Color Picker, which induced multiple image analysis methods that could be adjusted according to the intended measurements [6].

**Fig. 1:**
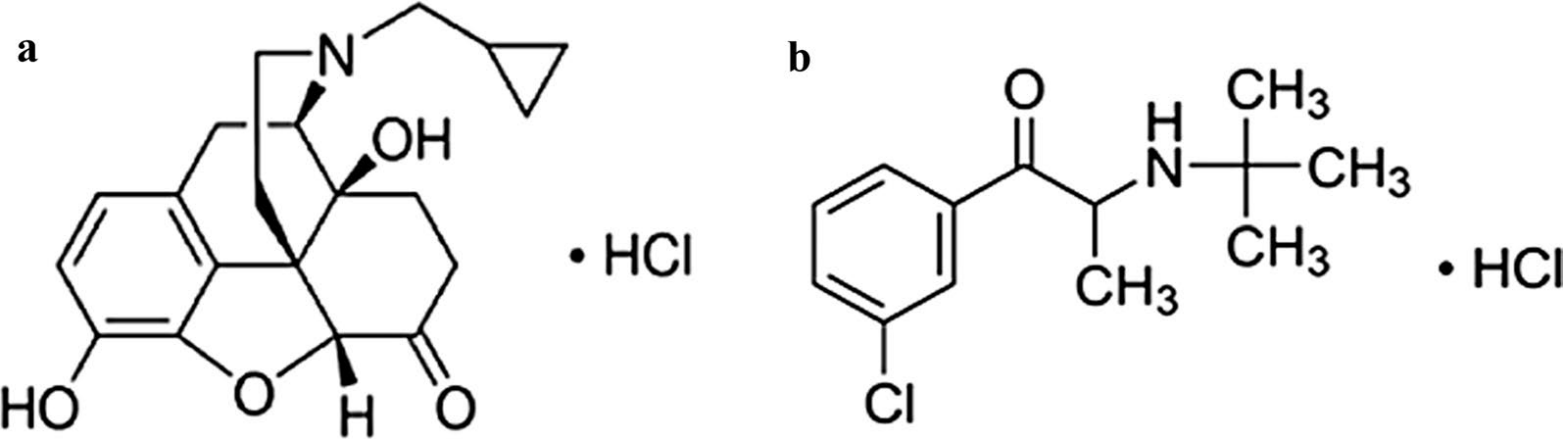
Structure of **a** NAL and **b** BUP

Naltrexone hydrochloride (NAL) (Fig. 1a) is an opioid receptor antagonist used in treating opioid addiction and alcoholism [10]. Bupropion hydrochloride (BUP) (Fig. 1b) is a norepinephrine and dopamine reuptake inhibitor, and also acts as a nicotinic receptor antagonist. It is prescribed for the management of major depressive disorders and aids in smoking cessation [11]. Both drugs act together in controlling energy balance and food intake, consequently, they are used in combination for obesity treatment [12]. The literature survey revealed two UV spectrophotometric articles including derivative spectroscopic, simultaneous equation, absorbance ratio, and dual wavelength methods [13, 14], and various high performance liquid chromatography (HPLC) methods [15–19] for the simultaneous assay for both drugs. There is always a need for simple, rapid, and cheap methods for quantitation of the studied drugs. As far as we know, no TLC approaches have been documented that address the same purpose.

The purpose of this work was to cultivate a green high performance thin layer chromatographic (HPTLC) method based on three detection techniques; namely a conventional densitometric measurement and two simple and accessible smartphone-dependent measurements for simultaneous analysis of NAL and BUP in their pure form and combined tablet formulation (Contrave®). The assessment of the content uniformity of dosage units was also performed. The methods were further evaluated by Green Analytical Procedure Index (GAPI) [20], Analytical GREEnness Metric Approach (AGREE) [21], and white analytical chemistry (WAC) approach [22]. The greenness assessment tool, GAPI, is a pictogram consisting of five divided pentagrams colored with red, yellow, or green stand for high, medium, and low hazardous effects, correspondingly. This tool evaluates the environmental outcome of the method’s steps starting from sample preparation steps and specification, reagents used regarding amounts and hazards, instrumentation and energy consumption up to waste amount and treatment [20]. The quantitative and schematic comparison presented by AGREE tool facilitated the assessment process by evaluating the compliance of each method’s steps to the 12 principles of green analytical chemistry and calculating a score from 0 to 1 with a clock-like colored pictogram signifying the level of greenness for each step and a central circle showing the calculated AGREE score, which becomes greener as the score approaches 1 [21]. The whiteness assessment accounts for the method’s analytical and practical performance in addition to its greenness. A Red Green Blue (RGB) 12 model was introduced to evaluate the sustainability and whiteness degree for the tested analytical methods through three divided groups each representing an assessment criterion by a rank and an assigned color. The red color represents the method’s analytical performance regarding the scope of application and validation parameters, the green color represents the method’s safety and environmental outcomes through covering the most prominent parameters of green analytical chemistry, while the blue one is representing the method’s practical and economic sides. The compound saturation of the three colors induces color shades from black/grey to white, in addition to quantitative evaluation for each parameter that also get combined together to give an overall numeric evaluation for the method from 0 to 100, where black shade and 0 rank for the least fitted method and white color with 100 rank for the best appropriate method [22].

## Experimental

### Material and Methods

#### Chemicals and reagents

NAL (BN: PDNRHNF002) was provided by Eva Pharma (Giza, Egypt) and BUP (BN: ACBUPNF016) was supplied by Sun Pharmaceutical Industries Ltd (Giza, Egypt). Their potencies were checked by a reported HPLC method [17] and were found to be 100.49 ± 1.99 and 100.08 ± 1.98, for NAL and BUP, respectively. Contrave® tablets (BN: E1687A) manufactured by Orexigen Therapeutics Inc. (California, USA) were purchased from the Canadian local market. Each tablet claimed to contain 8 mg of NAL and 90 mg of BUP. Analytical grade methanol, ethyl acetate, and sodium nitrite were acquired from Piochem Company (Giza, Egypt). Acetone was bought from ZI de Valdonne (Peypin, France). Glacial acetic acid and basic bismuth nitrate were obtained from Sigma Aldrich (MO, USA). Potassium iodide and sodium nitrite were purchased from El-Nasr Pharmaceutical Chemical Co. (Cairo, Egypt). Dragendorff’s reagent was prepared by mixing 70 mL distilled water and 20 mL acetic acid with 5 mL of 40 g% potassium iodide solution and 5 mL of 1.7 g% w/v basic bismuth nitrate in 20% v/v acetic acid solution [23].

#### Instruments and chromatographic conditions

The studied drugs were separated using HPTLC aluminum plates (20 × 20 cm, 0.1 mm) pre-coated with silica gel 60 F_254_, E. Merck (Germany). Samples were applied onto the plate as bands with lengths of approximately 6 mm separated by 4 mm and positioned 1.5 cm from the bottom edge of the plate. These procedures were using Camag Linomat 5 autosampler (Switzerland) along with Camag microsyringe (100-mL) and Camag software. Separation was reached by a developing system composed of ethyl acetate: methanol: acetone: glacial acetic acid in a ratio of 3:6.5:1.5:0.5, by volume. The system was allowed to saturate the jar for approximately 10 min before development. Samples were developed by ascending mode at ambient temperature in a glass TLC-tank.

For the densitometric method: The air-dried plates were scanned at 203 nm by a Camag scanner model 3S/N 130319 with slit dimensions (3 × 0.5mm) programmed with winCATS software at 20 mm/second scan speed.

For the smartphone method: In a lab fume hood, the developed plates were immersed in Dragendorff’s reagent for 30 s and left to dry for at least 5 min, then sprayed with 5% w/v sodium nitrite solution. The plates were kept at 20 cm distant from the sprayer. The plates turned to brown due to iodine formation, which faded with time leaving a light-yellow background with brown spots of NAL and BUP. The sprayed plates were left to dry for 5 min and placed in Lámpara UV DESAGA multi-purpose equipment for UV 254/366 nm and daylight illumination (Uruguay) covered with a cardboard box. Samsung Galaxy A70 rear 32 MP camera was used to capture images of the plates at a 15-cm distance under daylight illumination. The images were opened in ImageJ software version 153 (NIH, USA) on a laptop, in addition to the Color Picker smartphone free application version 7.6.3 (https://play.google.com/store/apps/details?id=gmikhail.colorpicker) for further quantitative measurements. Each opened image in ImageJ software was analyzed as reported [3]. Briefly, the rectangular selection tool was used to draw equal-sized rectangles to define each sample track, followed by numbering each lane by “Gels” menu in “Analyze” drop-down menu then “Plot Lanes” option was chosen. The generated peaks equivalent to each sprayed spot were manipulated by straight line and magic wand tools to calculate their corresponding peak areas. While in case of Color Picker application [6], for each image, the size of the aim shape (circle) was adjusted to surround the largest spot using the dropper-shaped button manually, then used to detect the luminance for each spot and the background as well.

### Solutions preparations

#### Standard stock solutions of NAL and BUP

Stock standard solutions of NAL and BUP (1 mg/mL) were prepared separately, by weighing 50 mg of each compound into a 50-mL measuring flask and completing the volume using methanol. The solutions were refrigerated at 8 ºC keeping them stable for up to a month.

Various calculated volumes were drawn from the stock standard solutions and transferred into a set of 10-mLvolumetric flasks, then the blue film coat, then weighted, grounded, and mixed well, and then powdered amounts equivalent to 8 mg NAL and 90 mg BUP were sonicated with 70 mL methanol for 30 min volumes were completed with methanol to prepare the laboratory mixtures.

#### Construction of calibration curves

Aliquots equivalent to 0.1 to 30 mg were transferred from the stock standard solutions into two individual sets of 10-mL measuring flasks and volumes were completed with methanol. From each solution, 10 µL was applied in triplicates on TLC silica plates and chromatographed as previously described. For the densitometric and the smartphone-ImageJ methods, the average peak areas obtained were plotted against the respective concentrations to develop the calibration curves, and the regression equations were calculated. While for the smartphone-Color Picker method, the average differences between the spots’ luminance and the background’s luminance were plotted instead of peak areas. Solutions for accuracy, precision, and robustness were prepared and the methods’ were validated consistent with ICH-guidelines [24].

### Application to pharmaceutical preparation analysis and content uniformity testing

Ten Tablets of Contrave® were subjected to whipping with cotton wetted with methanol to remove the blue film coat then were weighted, grounded and mixed well, and then amounts equivalent to 8 mg NAL and 90 mg BUP were sonicated with 70 mL methanol for 30 min, filtered through Whatman® filter paper (grade 1), and quantitatively transferred to 100-mL measuring flasks for direct tablet analysis. Additionally, for applying the standard addition technique, fixed amounts of tablet formulation corresponding to a certain concentration and variable standard powders (equivalent to half, equal, and double the tablet amount) were mixed for each drug, then extracted as previously stated.

The mentioned procedures were repeated for 10 tablets individually for the content uniformity testing by densitometric and smartphone-ImageJ methods and uniformity was evaluated according to the USP guidelines [25].

## Results and discussion

### Method development and optimization

#### Separation optimization

**Fig. 2:**
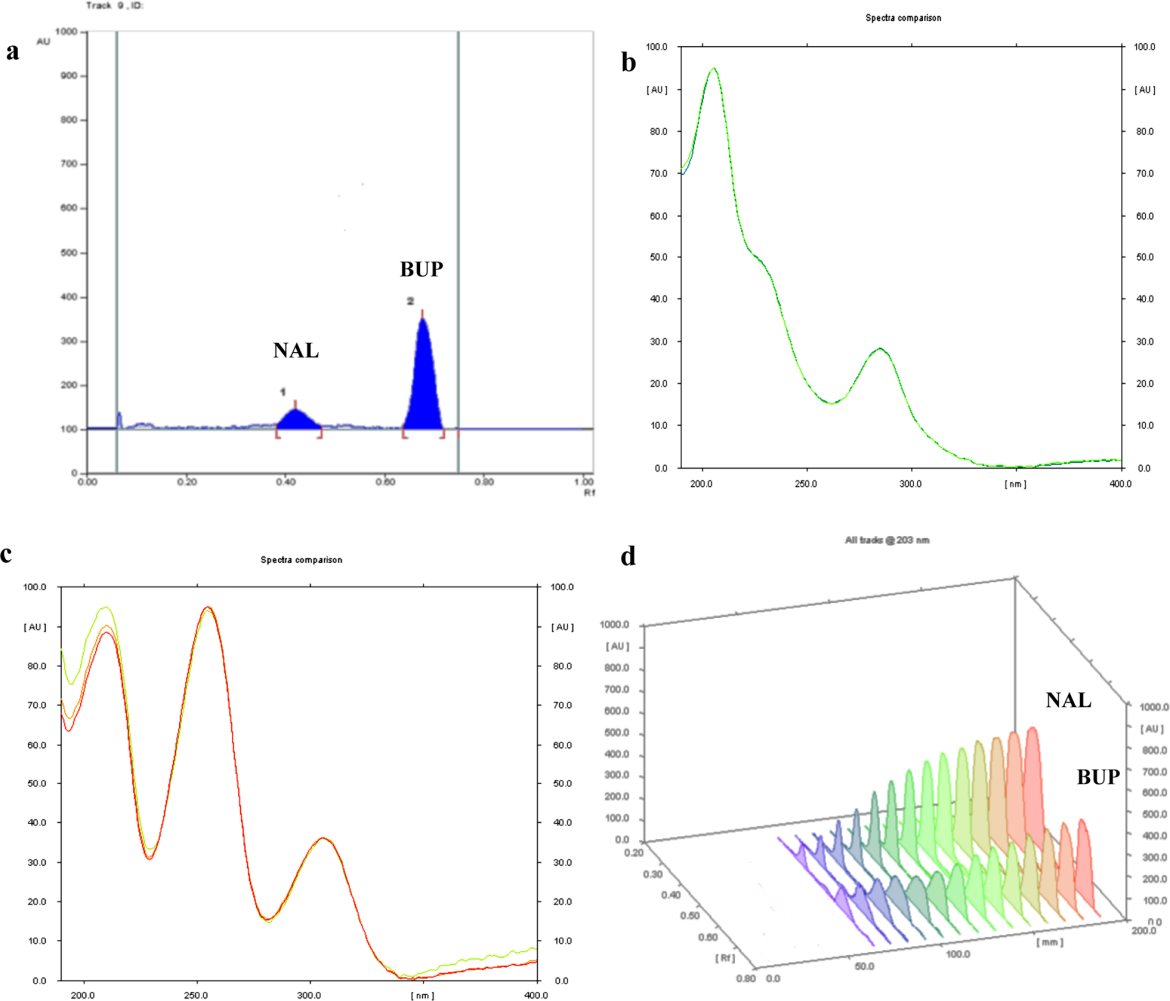
**a** HPTLC chromatogram of NAL (0.8 µg/band) and BUP (9 µg/band); **b**, **c** Spectrodensitograms of NAL and BUP peaks, respectively; **d** linearity range for NAL (0.4–24 µg/band) and BUP (0.6–18 µg/band)

TLC technique offers a well-established, simple, and economical method of analysis for numerous analytes with minimal solvent consumption per run [26, 27]. HPTLC plates offer improved peaks resolution and symmetry owing to their smaller particle size and thickness over classical TLC plates [28]. Different elution mixtures were tried using ethanol, methanol, butanol, isopropanol, ethyl acetate, and acetone. Utilizing ethanol or less polar alcohols eluted the drugs in the form of streaks rather than spots, while by adding acetone, an improvement of the symmetrical shape for the peaks was observed, especially BUP. However, due to the closeness of pKa values (NAL: 8.38 for the tertiary amine and 9.93 for the phenolic group, BUP: 8.35 for the secondary amine) [29], optimum resolution was achieved after the addition of either glacial acetic acid or ammonia solution. Acetic acid induced a superior effect on the peak symmetry of NAL and lowered the retardation factor (R_f_) for BUP peak away from the solvent front over ammonia. That may be attributed to the bond formed between the amine group in BUP and the polar silica of the stationary phase in the presence of acetic acid. Satisfactory results were obtained while using a mixture of ethyl acetate: methanol: acetone: acetic acid in a ratio of (3:6:1:0.5, by volume) at ambient temperature. The drugs were separated at R_f_ values of 0.41 and 0.67 for NAL and BUP, respectively (Fig. 2a). The bands were clearly distinguished with reasonable resolution. Symmetry and other system suitability parameters were calculated and presented in Table 1.

**Table 1:** System Suitability Parameters of the proposed densitometric method calculated as per ICH guidelines

	NAL		BUP
Retardation factor R_f_	0.41 ± 0.03		0.67 ± 0.03
Selectivity factor α^*^		2.922	
Resolution R_s_^*^		2.889	
Tailing factor T	1.125		1

^*^Calculated for each of two successive peaks

#### Quantitative measurements optimization

For the densitometric method: The spectrodensitograms of each drug were recorded after chromatographic separation (Fig. 2b, c). NAL spectrum showed maximum absorbance at a wavelength of 203 nm with reduced absorptivity at higher wavelengths, while BUP spectrum showed peak absorbance at 210 nm. The optimum scanning wavelength was chosen in preference of NAL on account of being the minor drug in the tablet dosage form (NAL: BUP; 8: 90), hence 203 nm was chosen for densitometric measurements. The concentration ranges were found to be 0.4–24 µg/band and 0.6–18 µg/band for NAL and BUP, respectively (Fig. 2d).

##### For smartphone methods

The application of smartphone camera offered a simple, portable, convenient, and economical alternative to complicated instruments and special software systems. However, to decrease possible variations from light, distance, and shooting parameters, some precautions were followed. The mentioned light source was kept in a cardboard box with a rectangular opening (4 × 2 cm) fitting to the UV lamps’ surface gap at a 15 cm distance from the plate position to eliminate external lights. Upon increasing the distance, a slight decrease in the image resolution was detected. To optimize the shooting conditions, camera mode was changed to pro mode instead of auto photo mode to standardize the following shooting parameters; ratio kept at (3:4 32 MP), flash was turned off, metering chosen to be center-weighted, and exposure was maintained (0.0). The white balance for daylight illumination showed suitable color temperature for the spots and background at 6000 k and the ISO sensitivity parameter was manually adjusted to 100, giving optimum sharpness and minimum noise.

**Fig. 3:**
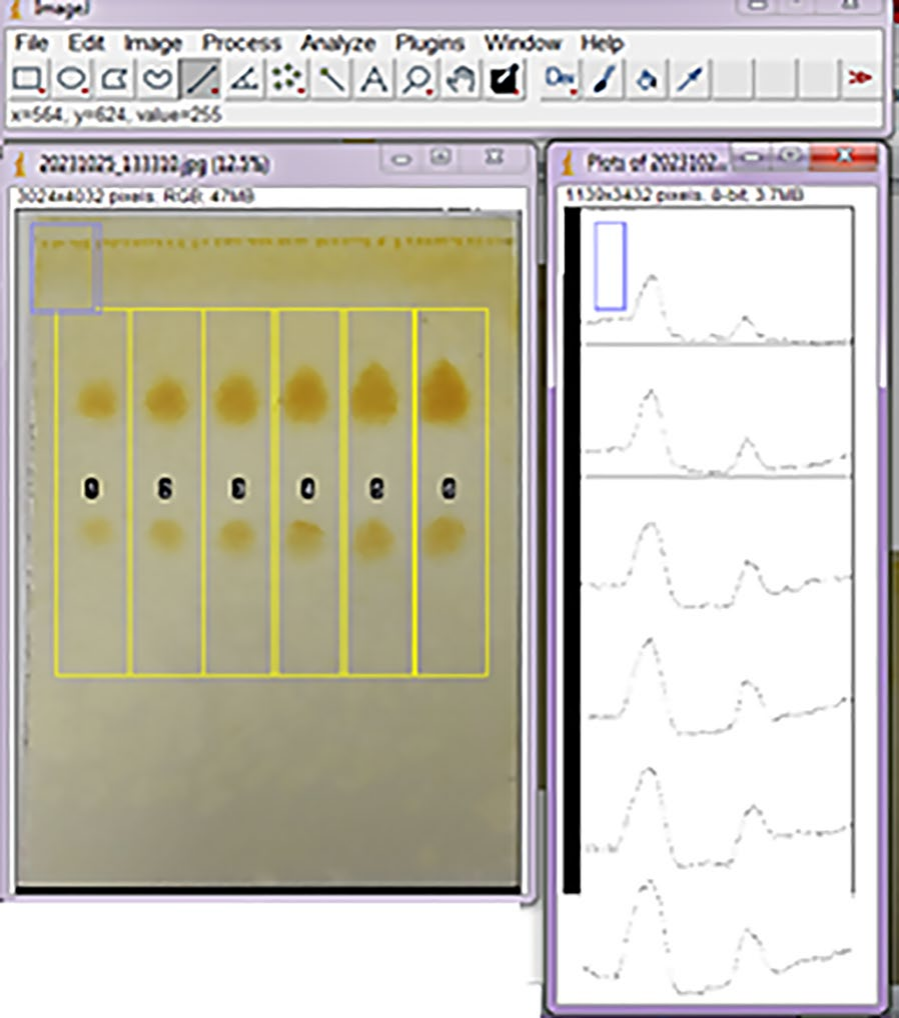
Quantification of visualized plates by ImageJ software

The poor absorptivity of NAL at 254.0 nm barred proper display under UV lamp light, therefore the utilization of staining reagent is essential. Iodine crystals were universally used for this purpose. However, the produced color intensity varied with time and faded rapidly. The presence of nitrogen atoms in the amine groups in both drugs suggested Marquis, ninhydrin, and Dragendorff’s reagents in addition to phosphomolybdic acid as alternative staining reagents. Marquis reagent contained harmful sulfuric acid and formaldehyde, ninhydrin and phosphomolybdic acid necessitated plate heating, therefore Dragendorff’s reagent was applied [23]. The heavy metals in Dragendorff’s reagent reacted with nitrogen in amine groups forming a colored precipitate; orange with tertiary amines, less color intensity with secondary amines. Immersing the eluted plates in Dragendorff’s reagent showed orange spots of the separated drugs, however, the sensitivity was insufficient compared with the densitometric method. Consequently, spraying the plates with sodium nitrite solution (5% w/v) after Dragendorff’s reagent (modified Dragendorff’s reagent) generated iodine that darkened the plates and intensified the spots’ color [5]. The Dragendorff’s reagent preference for tertiary amine in NAL structure in addition to nitrite spraying overcame the challenging detection of NAL with BUP in their combined dosage form ratio in optimal concentration ranges and spots shape (Fig. 3). The taken images were analyzed via ImageJ desktop software and Color picker mobile application as mentioned in the experimental section.

### Method validation

The developed methods were fully validated in terms of linearity range, limit of detection (LOD), limit of quantification (LOQ), accuracy, precision, specificity, robustness, and system suitability parameters per ICH guidelines [24] (Tables 1, 2).

**Table 2:** Validation parameters for the proposed methods

Item	TLC-Densitometric		TLC-ImageJ		TLC-Color Picker	
	NAL	BUP	NAL	BUP	NAL	BUP
Range (µg/band)	0.4–24	0.6–18	0.4–24	2–24	0.8–20	5–20
Slope (b)^a^	0.177	0.272	0.158	0.157	0.404	0.619
Intercept (a)^a^	0.138	0.277	0.257	0.611	0.923	-1.372
Correlation coefficient (r)	0.99965	0.9997	0.99995	0.9992	0.9972	0.9964
Accuracy (Mean ± SD)	101.85 ± 1.35	100.47 ± 1.47	99.61 ± 1.34	98.51 ± 1.03	100.04 ± 1.89	100.64 ± 1.87
Precision (%RSD)^b^ (%RSD)^c^						
	1.62	1.21	1.31	1.19	1.58	1.28
	1.65	1.68	1.73	1.80	1.93	1.92
Specificity (Mean ± SD)^d^	100.75 ± 1.72	99.78 ± 1.81	99.87 ± 1.85	100.58 ± 1.07	101.15 ± 1.67	100.96 ± 1.78
Robustness (%RSD)^e^	1.86	1.40	1.93	1.06	1.65	1.76
LOD (µg/band)	0.1	0.2	0.1	0.6	0.1	0.6
LOQ (µg/band)	0.3	0.6	0.3	2	0.8	5

^a^Regression equation for HPLC: A = a ‏ + bC, where ‘A’ is the area and ‘C’ is the concentration of NAL and BUP

^b^Intraday precision [average of three different concentrations of three replicate each (n = 9) within the same day]

^c^Interday precision [average of three different concentrations of three replicate each (n = 9) repeated on three successive days]

^d^Recovery of NAL and BUP in laboratory prepared mixtures

^e^Robustness; RSD, % (average of three different concentrations of three replicate each (n = 9) analyzed in different conditions mentioned before)

#### Linearity range

##### Densitometric method

The linearity ranges of each of the two drugs were found to be 0.4–24 µg/band and 0.6–18 µg/band for NAL and BUP, respectively, while LOD and LOQ were calculated by signal to noise ratio method and were found to be 0.1 & 0.3 µg/band for NAL and 0.2 & 0.6 µg/band for BUP, respectively (Table 2).

##### Smartphone methods

*ImageJ software*: The calculated peak areas of each of the two drugs were plotted against their corresponding concentration and linearity ranges were found to be 0.4–24 µg/band and 2–24 µg/band NAL and BUP, respectively, also LOD and LOQ were calculated by signal to noise ratio method and were found to be 0.1 & 0.3 µg/band for NAL and 0.6 & 2 µg/band for BUP, respectively (Table 2).

-*Color picker application:* The linearity ranges were determined by plotting the average of differences between the luminance of spots and the background against the matching spots’ concentrations. This way of plotting luminance differences instead of spots’ luminance against concentrations overcame the variations of circle tool’s sizes between different images, possible minor deviations of the light fallen on the plates or potential patchy plate drying. Additionally, it gave a direct proportional correlation with the measured concentrations. The linearity ranges were found to be 0.8–20 µg/band and 5.0–20 µg/band NAL and BUP, respectively, moreover LOD and LOQ were calculated by visual evaluation method and were found to be 0.1 & 0.8 µg/band for NAL and 0.6 & 5 µg/band for BUP, respectively (Table 2).

#### Accuracy and precision

The accuracy of the proposed methods was tested by assaying three varying concentrations within the linearity ranges three times and computing their mean recoveries percentage for each drug. While their precisions were evaluated in three different concentrations three times on three plates on the same day for intraday repeatability and on three consequent days for interday reproducibility (Table 2).

#### Robustness

Deliberate alterations of the conditions of the proposed methods were individually performed to estimate their reliability with minimum changes in normal usage. The mobile phase ratio changed with value of ± 0.2 for ethyl acetate, methanol, and acetone and saturation time changed to 15 min. Additionally, for the densitometric method, the scanning wavelength changed to 205 nm, and for smartphone methods, the images were captured at 20 cm distance. Peaks were well separated with unremarkable changes in methods’ parameters and accepted relative standard deviations in all performed alterations (Table 2).

#### Specificity

The Specificity of the methods was evaluated by analyzing both drugs in presence of each other in different concentrations percentages. NAL and BUP were mixed and analyzed by the suggested methods and the results demonstrated that both drugs could be determined together without interference (Table S1).

### Analysis of pharmaceutical formulation

Both drugs were analyzed in their combined dosage form with the co-formulated excipients by direct assay of the tablets’ solutions and by employing the standard addition technique. Concentrations were calculated using the obtained regression equation and the mean percentages recovery were presented as shown in Table S2.

#### Content uniformity testing

Ten tablets were individually treated and analyzed as mentioned in the densitometric and smartphone-ImageJ software methods according to the USP guidelines [25]. The mean recovery percentage (X^\^), standard deviation (SD), and percentage relative standard deviation (%RSD) were computed. The acceptance value was calculated by the formula (AV =|M–X^\^|+ K SD), where “K” is the acceptability constant which is equal to 2.4 when the number of tested units is 10, “M” is the reference value, that is equal to X^\^ when (98.5 ≤ X^\^ ≤ 101.5). The AV was found to be less than the maximum allowed AV (L1) representing a uniform distribution of both drugs in their combined dosage form (Table S3).

### Statistical evaluation of the proposed methods

The proposed methods were statistically compared against a reported HPLC method [17] for both drugs. The calculated student’s *t*-test and *F*-value with 95% confidence level indicated that there were no significant differences between the proposed and reported methods in terms of accuracy and precision (Table 3). Additionally, the sensitivity and linearity of the proposed methods were compared with that of the reported methods and represented in Table 3 and supplementary table S4.

**Table 3:** Statistical comparison of the results obtained by the proposed methods and the reported one [17]

	NAL				BUP			
Value	Densitometry	ImageJ	Color Picker	Reported^a^	Densitometry	ImageJ	Color Picker	Reported^a^
Mean	100.18	100.98	100.37	100.08	100.11	99.68	100.49	99.86
SD	1.92	1.86	1.97	1.98	1.89	1.93	1.99	1.63
%RSD	1.92	1.84	1.96	1.98	1.89	1.94	1.98	1.63
Variance	3.69	3.46	3.88	3.92	3.57	3.73	3.96	2.66
N	7	7	5	5	7	7	5	5
t-test^b^	0.087 (2.228)	0.796 (2.228)	0.232 (2.306)		0.245 (2.228)	0.175 (2.228)	0.548 (2.306)	
F Value^b^	1.063 (4.530)	1.133 (4.530)	1.010 (6.390)		1.344 (4.530)	1.402 (4.530)	1.490 (6.390)	
Linearity*	0.4–24	0.4–24	0.8–20	6.25–18.75	0.6–18	2–24	5–20	37.5–112.5
LOD*	0.1	0.1	0.1	NA	0.2	0.6	0.6	NA
LOQ*	0.3	0.3	0.8	NA	0.6	2	5	NA

*NA* Not available

^a^RP-HPLC method with mobile phase composed phosphate buffer (pH 3) and acetonitrile in ratio of 60: 40. The flow rate was adjusted at 1 mL/min and UV detection at 224 nm

^b^The values in the parenthesis are the corresponding theoretical values of t and F at P = 0.05

*Units in (µg/band) for the proposed methods and in (µg/mL) for the reported one

### Green profile and whiteness assessment metrics

The establishment of environmentally friendly methods has become a cornerstone in the analytical field [27, 30, 31]. The metric tools GAPI, and AGREE, in addition to WAC tool were performed to assess the greenness and sustainability of the proposed methods compared with reported methods (Table 4, Table S4).

**Table 4:** Green and white assessment comparison between the proposed and the reported methods

	Densitometric method	Smartphone methods	Reported method [17]
GAPI			
AGREE			
WAC			

GAPI results revealed that, the proposed methods showed greener superiority regarding sample treatment over the reported HPLC methods, also the smartphone methods offered less energy consumption advantage compared with the densitometric one.

AGREE results revealed that, the proposed methods gave higher scores owing to minimal sample treatment, waste amount per sample, and number of samples analyzed per hour, additionally, the less energy consumption and capability of at-line analysis in the smartphone methods elaborated their AGREE score over the densitometric method.

Also, the whiteness assessment results reveal the highest overall rank was for the densitometric method, especially for the analytical performance and validation parameters. The smartphone methods were greener in terms of energy consumption and higher in practical and cost aspects regarding the elimination of the expensive detector which has slightly complicated adjustment steps, although the densitometric method was more time efficient due to the simultaneous integration of the results.

The reported HPLC methods are offline high-cost techniques that consume high energy and require micro-syringe filtration for the samples as well as filtration for the buffer part of the mobile phase, well-trained personal staff, and complicated and costly instruments, so they showed the least greenness and whiteness assessment results compared with the proposed methods.

As presented, the proposed methods showed suitable performance. The measurements using Color Picker application were incapable of distinguishing between minimal variations in analytes concentrations, so this method wasn’t applied in content uniformity testing. Comparing the validation results and the greenness and whiteness assessment results discovered the closeness between the densitometric method and smartphone using ImageJ software method concerning greenness and sustainability followed by smartphone using Color Picker application method.

## Conclusion

In this work, new HPTLC methods were reported for assaying NAL and BUP in their combined dosage form. The work involved the use of a smartphone camera integrated with either image processing software or mobile application as simple and economical alternatives to conventional densitometric measurements. The developed methods were validated according to the ICH guidelines, statistically compared to a reported HPLC method, and assessed by three metric tools for greenness and sustainability. The results confirmed the capability of the proposed methods to be used for the routine analysis of the separated drugs in their pure forms and combined pharmaceutical formulation. Although the densitometric method showed slightly higher analytical performance, the smartphone-based methods were more effective regarding fair cost, portability and handling effort, work experience, laboratory requirements, and energy consumption. The proposed methods confirmed the capability of the smartphone technologies to displace conventional, expensive, and intransigent methods. The developed methods have a positive impact on low-budget facilities by enabling them to utilize affordable and widely accessible technologies in routine analysis.

## Supplementary Information

Supplementary Material 1

## Data Availability

The data used and/or analyzed during this study are available from the corresponding author on a reasonable request.

## Abbreviations

AGREE: Analytical GREEnness Metric Approach
AV: Acceptance Value
BUP: Bupropion Hydrochloride
GAPI: Green Analytical Procedure Index
HPTLC: High Performance Thin Layer Chromatographic
ICH: International conference of harmonization
LOD: Limit of detection
LOQ: Limit of Quantification
NAL: Naltrexone Hydrochloride
NIH: National Institute of Health
USP: United States Pharmacopeia
