# Supplementary material for: An eco-friendly smartphone based HPTLC method versus conventional densitometric one for determination of Naltrexone and Bupropion
Source: BMC Chem. 2024 Sep 23;18(1):185. doi: 10.1186/s13065-024-01285-1 (PMC11421204; doi:10.1186/s13065-024-01285-1)
Supplement: Supplementary file 1 — Supplementary Material 1 [file 13065_2024_1285_MOESM1_ESM.docx]

**An Eco-friendly Smartphone based HPTLC Method *versus* Conventional Densitometric One for Determination of Naltrexone and Bupropion**

Eman M. Moaaz,* Ezzat M. Abdel-Moety, Mamdouh R. Rezk, Ahmed S. Fayed

*Analytical Chemistry Department, Faculty of Pharmacy-Cairo University, Kasr El-Aini Street, ET-11562 Cairo, Egypt*

* Corresponding author email: [eman.moaaz@pharma.cu.edu.eg](mailto:eman.moaaz@pharma.cu.edu.eg)

**Supplementary Table S1.** Determination of NAL and BUP in laboratory prepared mixtures.

| Ratio | Conc. (µg/band) | | R% | | | | | |
| --- | --- | --- | --- | --- | --- | --- | --- | --- |
|  | **NAL** | **BUP** | **TLC-Densitometry** | | **TLC-ImageJ** | | **TLC-Color Picker** | |
|  |  |  | **NAL** | **BUP** | **NAL** | **BUP** | **NAL** | **BUP** |
| 8:90 | 0.8 | 9 | 99.76 | 99.11 | 98.95 | 99.47 | 101.26 | 102.31 |
| 10:90 | 1 | 9 | 97.25 | 97.06 | 100.35 | 101.75 | 100.82 | 101.77 |
| 6:90 | 0.6 | 9 | 102.62 | 98.29 | 97.67 | 100.13 | - | 98.18 |
| 8:80 | 0.8 | 8 | 100.32 | 102.29 | 100.92 | 102.27 | 104.35 | 101.78 |
| 8:100 | 0.8 | 10 | 100.81 | 100.60 | 98.16 | 99.37 | 98.16 | 98.05 |
| 8:90 | 0.8 | 9 | 101.45 | 101.15 | 99.74 | 101.60 | 103.11 | 101.77 |
| 10:90 | 1 | 9 | 103.62 | 101.56 | 100.03 | 100.12 | 101.81 | 98.72 |
| 6:90 | 0.6 | 9 | 100.46 | 98.29 | 103.46 | 100.81 | - | 101.05 |
| 8:80 | 0.8 | 8 | 101.31 | 98.15 | 101.71 | 101.02 | 101.26 | 100.36 |
| 8:100 | 0.8 | 10 | 99.97 | 101.34 | 97.76 | 99.31 | 99.40 | 102.90 |
| (Mean±SD) |  |  | 100.75±1.72 | 99.78±1.81 | 99.87±1.85 | 100.58±1.07 | 101.27±1.95 | 100.96±1.78 |
| %RSD |  |  | 1.71 | 1.81 | 1.85 | 1.06 | 1.92 | 1.76 |

**Supplementary Table S2.** Determination of NAL and BUP in their combined pharmaceutical dosage forms by the proposed methods and application of the standard addition technique.

| Product |  | Drug | Drug Product | Standard addition^*^ | | | |
| --- | --- | --- | --- | --- | --- | --- | --- |
| Contrave^®^  NAL/BUP  8/90mg |  |  | **R%±SD** | **Taken (µg/band)** | **Added (µg/band)** | | **R%** |
|  | **Densitometry** | **NAL** | 100.14±1.09 | 0.8 | 0.4 | | 97.74 |
|  |  |  |  |  | 0.8 | | 99.30 |
|  |  |  |  |  | 1.2 | | 99.87 |
|  |  |  |  | **Mean±SD** | | | **98.97±1.11** |
|  |  | **BUP** | 100.41±1.31 | 4 | 2 | | 100.16 |
|  |  |  |  |  | 4 | | 99.99 |
|  |  |  |  |  | 6 | | 100.06 |
|  |  |  |  | **Mean±SD** | | | **100.09±0.09** |
|  | **ImageJ** | **NAL** | 98.81±1.25 | 0.8 | | 0.4 | 99.75 |
|  |  |  |  |  |  | 0.8 | 100.12 |
|  |  |  |  |  |  | 1.2 | 100.02 |
|  |  |  |  | **Mean±SD** | | | **99.96±0.19** |
|  |  | **BUP** | 100.61±1.71 | 4 | | 2 | 100.75 |
|  |  |  |  |  |  | 4 | 100.16 |
|  |  |  |  |  |  | 6 | 100.27 |
|  |  |  |  | **Mean±SD** | | | **100.39±0.31** |
|  | **Color Picker** | **NAL** | 98.76±1.78 | 0.8 | | 0.4 | 98.46 |
|  |  |  |  |  |  | 0.8 | 100.16 |
|  |  |  |  |  |  | 1.2 | 97.46 |
|  |  |  |  | **Mean±SD** | | | **98.9±1.36** |
|  |  | **BUP** | 101.71±1.15 | 4 | | 2 | 99.63938 |
|  |  |  |  |  |  | 4 | 101.7762 |
|  |  |  |  |  |  | 6 | 103.2402 |
|  |  |  |  | **Mean±SD** | | | **101.55±1.81** |

^*^Average of three determination.

**Supplementary Table S3.** Content uniformity testing’s results for determination of NAL and BUP in Contrave^®^ tablets by TLC-densitometric method and TLC-ImageJ method.

| Tab. No. | Tab. Weight (mg) | R% | | | |
| --- | --- | --- | --- | --- | --- |
|  |  | **TLC-Densitometry** | | **TLC-ImageJ** | |
|  |  | **NAL** | **BUP** | **NAL** | **BUP** |
| 1 | 696.4 | 99.83 | 99.11 | 99.10 | 101.53 |
| 2 | 689.5 | 101.10 | 99.77 | 97.76 | 104.33 |
| 3 | 687.1 | 100.25 | 103.60 | 100.92 | 99.42 |
| 4 | 689.7 | 101.80 | 97.06 | 104.88 | 100.20 |
| 5 | 688.9 | 103.92 | 98.29 | 101.24 | 101.10 |
| 6 | 690.1 | 97.22 | 99.47 | 101.64 | 100.39 |
| 7 | 694.2 | 104.34 | 102.79 | 102.90 | 98.77 |
| 8 | 689.4 | 96.72 | 99.52 | 101.87 | 102.01 |
| 9 | 685.8 | 102.44 | 101.15 | 98.947 | 98.89 |
| 10 | 692.5 | 100.67 | 100.33 | 99.74 | 99.45 |
| Mean | 690.36 | 100.83 | 100.11 | 100.90 | 100.61 |
| SD | 3.19 | 2.51 | 1.97 | 2.10 | 1.71 |
| %RSD | 0.46 | 2.49 | 1.97 | 2.08 | 1.70 |
| AV^*^ |  | **6.024** | **4.728** | **5.04** | **4.104** |

^*^Acceptance value = 2.4 × SD.
Maximum allowed AV (L1) = 15.

**Supplementary Table S4** Comparison between proposed and reported methods regarding sensitivity, greenness, and whiteness.

| **Parameter** | **Densitometric method** | **Smartphone methods** | **UV method [13]** | **UV method [14]** | **HPLC method [15]** | **HPLC method [16]** | **HPLC method [17]** | **HPLC method [18]** | **HPLC method [19]** |
| --- | --- | --- | --- | --- | --- | --- | --- | --- | --- |
| **Linearity*** | NAL: 0.4-24  BUP: 0.6-18 | NAL:  IJ: 0.4-24  CP: 0.8-20  BUP:  IJ: 2-24  CP: 5-20 | NAL: 1-6  BUP: 10-60 | 2-10 for both | 40-100 for both | NAL: 40-200  BUP: 10-50 | NAL: 6.25-18.75  BUP: 37.5-112.5 | NAL: 10-90  BUP: 5-40 | NAL: 0.5-15  BUP: 1.2-18 |
| **LOD*** | NAL: 0.1  BUP: 0.2 | NAL: IJ: 0.1 /CP: 0.1  BUP: IJ: 0.6 /CP: 0.6 | NAL: 1.345  BUP: 1.611 | NAL: 0.13  BUP: 0.11 | 0.5 for both | NAL: 0.326  BUP: 0.436 | NA | NAL: 0.000046  BUP: 0.0068 | NAL: 0.1  BUP: 0.31 |
| **LOQ*** | NAL: 0.3  BUP: 0.6 | NAL: IJ: 0.3 /CP: 0.8  BUP: IJ: 2 /CP: 5 | NAL: 4.078  BUP: 4.833 | NAL: 0.39  BUP: 0.35 | 1.7 for both | NAL: 0.99  BUP: 1.321 | NA | NAL: 0.0205  BUP: 0.0208 | NAL: 0.3  BUP: 0.93 |
| **GAPI** | 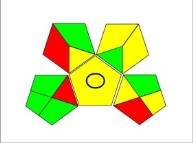 | 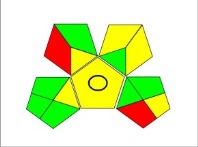 | 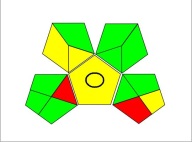 | 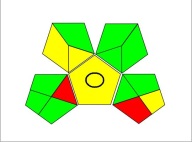 | 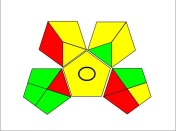 | 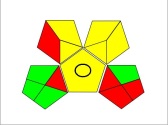 | 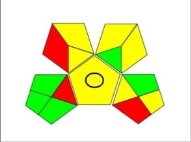 | 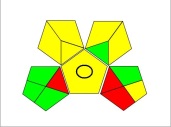 | 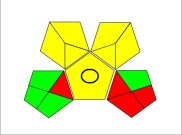 |
| **AGREE** | 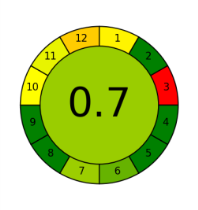 | 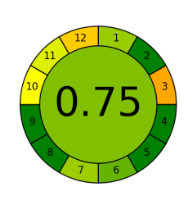 | 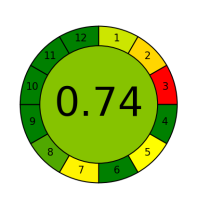 | 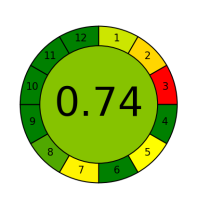 | 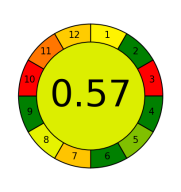 | 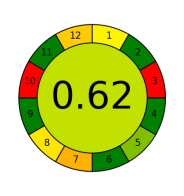 | 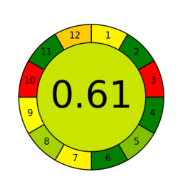 | 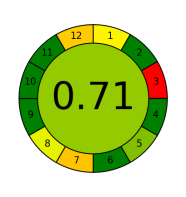 | 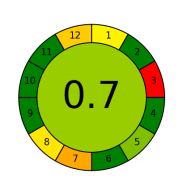 |
| **WAC** | 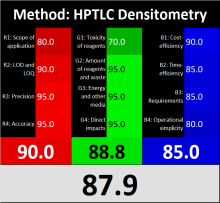 | 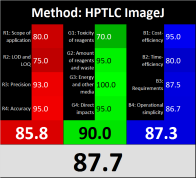  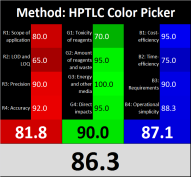 |  |  |  |  | 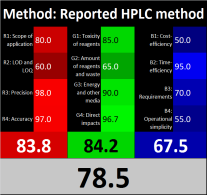 |  |  |

*Units in (µg/band) for the proposed methods and in (µg/mL) for the reported methods

IJ: ImageJ – CP: Color Picker - NA: Not available
